# Potential mitochondria-associated pathogenic genes in sepsis: a multi-omics Mendelian randomization study

**DOI:** 10.3389/fimmu.2026.1881375

**Published:** 2026-08-20

**Authors:** Lu Wang, Zhen Gao, Chengjin Wang, Yan Zhao, Zihui Deng, Yang Bai, Mengmeng Yang, Yuhang Zou, Hongjun Kang

**Affiliations:** 1 Department of Critical Care Medicine, the Fifth Medical Center, Chinese People’s Liberation Army (PLA) General Hospital, Beijing, China; 2 Chinese People’s Liberation Army (PLA) Medical School, Beijing, China; 3 Department of Critical Care Medicine, the First Medical Center, Chinese People’s Liberation Army (PLA) General Hospital, Beijing, China; 4 Department of Basic Medicine, Graduate School, Chinese People’s Liberation Army (PLA) General Hospital, Beijing, China; 5 Department of Emergency Medicine, the First Medical Center, Chinese People’s Liberation Army (PLA) General Hospital, Beijing, China; 6 National Key Laboratory of Kidney Diseases, National Clinical Research Center for Kidney Diseases, Beijing Key Laboratory of Kidney Disease Research, Beijing, China

**Keywords:** colocalization, Mendelian randomization, mitochondria, multi-omics, sepsis

## Abstract

**Background:**

Mitochondrial dysfunction has been implicated in the pathophysiology of sepsis. However, human genetic evidence linking mitochondria-related genes to sepsis susceptibility remains limited. This study aimed to identify mitochondria-related genes associated with sepsis risk using a multi-omics Mendelian randomization framework.

**Methods:**

Summary-data-based Mendelian randomization (SMR) was applied using sepsis genome-wide association study (GWAS) summary statistics from the UK Biobank and FinnGen databases. Expression, methylation, single-cell, and protein quantitative trait loci (QTLs) were used as genetic instruments. Colocalization analyses were conducted to evaluate whether SMR associations were driven by shared genetic variants. Expression of prioritized candidate genes was further examined in clinical septic samples, and correlations with disease severity (SOFA scores) were assessed.

**Results:**

SMR analysis prioritized 13 mitochondria-related genes associated with sepsis risk. Immune cell-specific eQTL analysis suggested that genetically predicted *SURF1* expression in memory B cells and naïve T cells was associated with sepsis risk. Differential expression of 12 candidate genes was confirmed in septic patients by qPCR, and *PPOX* expression showed a negative correlation with SOFA scores. Integration of mQTL and eQTL data supported a regulatory relationship between methylation at cg06661924 and *AK4* expression. Increased genetically predicted AK4 expression was associated with higher sepsis risk (OR = 1.21, 95% CI 1.02-1.42). Protein-level analysis identified DUT as a potential sepsis-associated candidate, with consistent evidence across streptococcal and pneumococcal septicemia subtypes. Subtype analyses also suggested heterogeneous genetic signals across different sepsis subtypes.

**Conclusion:**

This study prioritized several mitochondria-related genes associated with sepsis susceptibility based on human genetic evidence. These findings provide candidate targets for further mechanistic and translational investigation.

## Background

Sepsis is a life-threatening syndrome arising from a dysregulated host response to infection and frequently leading to acute organ dysfunction. It represents a major global health burden. Epidemiological analyses estimate that more than 30 million people develop sepsis each year and that the condition contributes to nearly six million deaths worldwide (1, 2). Clinically, sepsis is characterized by systemic inflammation, circulatory instability, and progressive organ failure when not recognized and treated promptly (2). Individuals of advanced age, patients with chronic illnesses such as diabetes or cardiovascular disease, those with impaired immunity, and individuals with repeated hospital exposure are particularly vulnerable to severe infection and subsequent sepsis (2). Early treatment with antimicrobial therapy, fluid resuscitation, and vasopressor support can improve outcomes, yet delayed diagnosis and intervention remain common and are associated with increased mortality (3). Despite substantial advances in intensive care medicine, sepsis continues to impose a heavy clinical burden. One major obstacle is the pronounced biological heterogeneity observed across patients, which complicates the identification of reliable molecular markers and broadly effective therapeutic targets.

Mitochondria play a central role in cellular energy metabolism and have increasingly been implicated in the pathophysiology of sepsis. These double-membrane organelles generate adenosine triphosphate (ATP) through oxidative phosphorylation and regulate multiple aspects of cellular homeostasis (4). Impairment of mitochondrial function can reduce ATP production and promote excessive reactive oxygen species generation, thereby disrupting redox balance and damaging cellular structures (5). Consistent with this, mitochondrial dysfunction-associated ferroptosis, an iron-dependent form of regulated cell death driven by lipid peroxidation, has been shown to contribute to sepsis-induced cardiac injury (6). Accumulating evidence indicates that mitochondrial dysfunction is closely associated with the progression of sepsis (7, 8). Experimental models have shown that systemic infection can impair mitochondrial respiration and bioenergetic capacity, suggesting that mitochondrial injury contributes to the development of organ dysfunction in septic states (8). In addition to disturbances in energy metabolism, mitochondrial signaling pathways have also been linked to inflammatory regulation, oxidative stress responses, and immune cell function during sepsis (7). These findings collectively highlight the potential importance of mitochondrial pathways in sepsis biology. Nevertheless, most available evidence originates from experimental systems or small clinical cohorts, and the contribution of mitochondrial genes to sepsis susceptibility at the population level remains incompletely understood.

Human genetic approaches offer an opportunity to investigate such relationships in a less confounded framework. Mendelian randomization (MR) uses genetic variants as instrumental variables to explore potential causal relationships between biological traits and disease outcomes, thereby reducing confounding that commonly affects observational studies (9). Building on this principle, summary-data-based Mendelian randomization (SMR) integrates genome-wide association study (GWAS) signals with molecular quantitative trait loci (QTL) data to prioritize genes whose molecular regulation may influence complex traits (10). The HEIDI test further evaluates whether observed associations are likely driven by a shared causal variant rather than linkage disequilibrium between nearby loci (11). By integrating genetic association signals with molecular regulatory data, this framework provides a systematic strategy for connecting disease-associated loci with candidate functional genes.

In the present study, we applied a multi-omics SMR framework to prioritize candidate mitochondria-related genes and regulatory loci that may causally contribute to sepsis susceptibility, thereby generating a shortlist of functional targets for future mechanistic investigation. Sepsis genetic association data were drawn from the UK Biobank, a population-based cohort of predominantly European ancestry (12), with independent replication using the Finnish FinnGen resource (13). Mitochondrial gene candidates were curated from established mitochondrial gene resources and interrogated using large-scale molecular QTL datasets spanning gene expression, DNA methylation and protein abundance, predominantly derived from populations of European ancestry, together with single-cell regulatory variation captured in the OneK1K cohort of 982 European-ancestry donors recruited in Australia (14). Signals supported by SMR and colocalization evidence were prioritized as candidate genes. To enhance biological relevance, genetically predicted effects were subsequently evaluated in clinical samples from septic patients, enabling comparison between predicted regulatory directions and observed gene expression patterns as well as their correlation with disease severity assessed by SOFA scores.

## Materials and methods

### Study design

**Figure 1:**
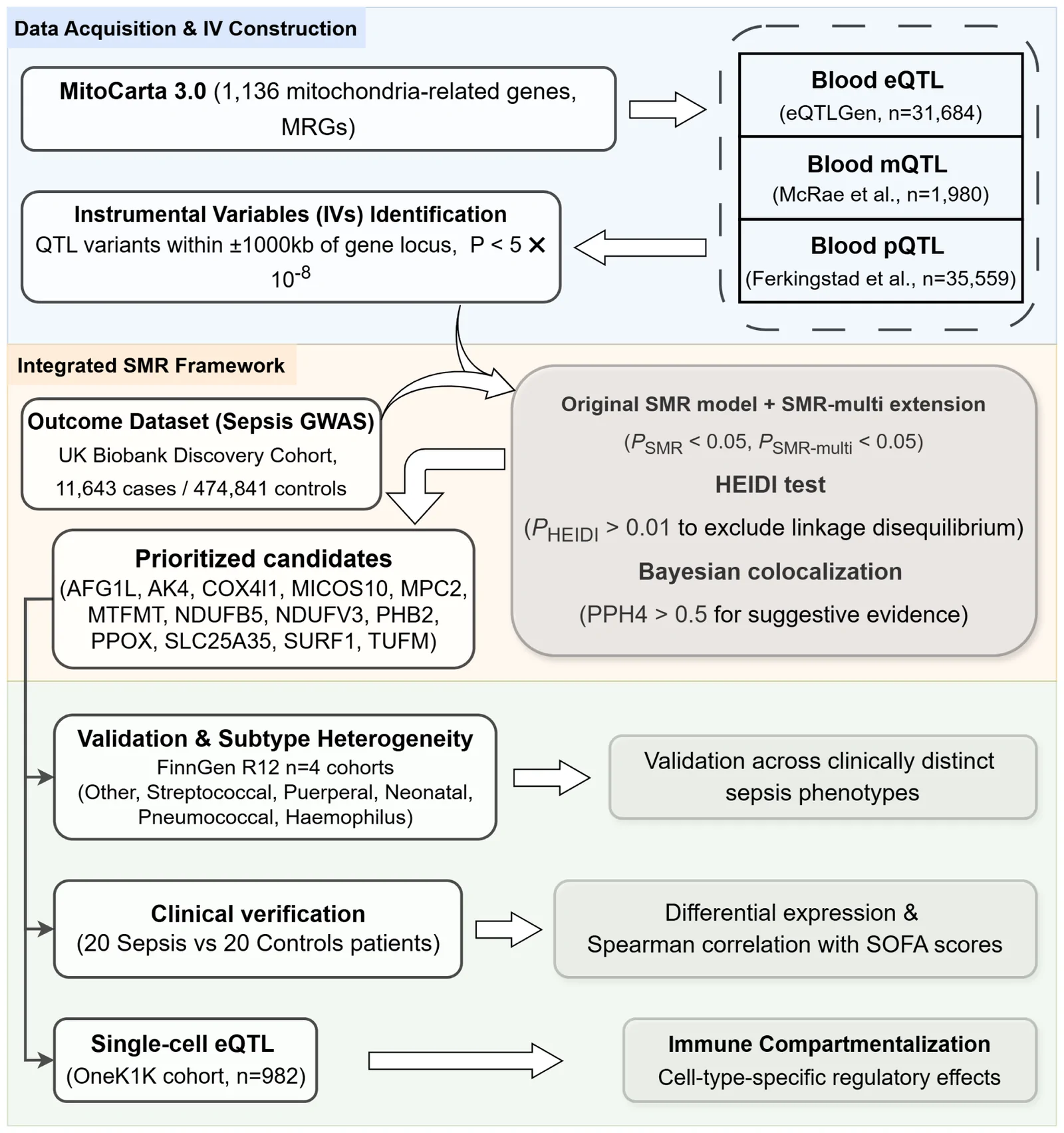
Study design and analytical framework. Overview of the multi-omics summary-data-based Mendelian randomization (SMR) pipeline used to prioritize mitochondria-related genes associated with sepsis. Genome-wide association study (GWAS) summary statistics for sepsis were integrated with multiple molecular quantitative trait loci (QTL) datasets, including blood expression QTLs (eQTLs), methylation QTLs (mQTLs), protein QTLs (pQTLs), and single-cell eQTLs. The analytical framework incorporates the SMR test, the heterogeneity in dependent instruments (HEIDI) test, and Bayesian colocalization analysis. Downstream analyses include clinical validation using quantitative real-time PCR (qPCR), correlation with disease severity measured by Sequential Organ Failure Assessment (SOFA) scores, subtype heterogeneity analyses in FinnGen cohorts, and immune cell compartment–specific regulatory analyses.

The overall analytical framework of this study is illustrated in **Figure 1**. A multi-layer integrative analysis strategy based on summary-data-based Mendelian randomization (SMR) was applied to investigate mitochondria-related genes potentially associated with sepsis susceptibility. Genome-wide association study (GWAS) summary statistics for sepsis from the UK Biobank (UKB) were used as the primary discovery dataset. Independent cohorts derived from previously published GWAS studies and the FinnGen project were further incorporated to evaluate the consistency of the observed associations. Genetic variants influencing molecular traits related to mitochondrial biology were used as instrumental variables, enabling the integration of molecular quantitative trait loci (QTL) signals with disease GWAS signals. Associations were systematically assessed across several biological layers, including DNA methylation, gene expression, single-cell expression, and circulating protein levels. This design allowed the investigation of potential regulatory relationships from epigenetic variation to transcriptional and proteomic alterations. To strengthen the interpretation of genetically supported associations, Bayesian colocalization analyses were subsequently performed to determine whether the molecular QTL signals and sepsis GWAS signals within a locus were likely to share a causal variant. The exposure and outcome datasets used in the SMR analyses were derived from independent populations, thereby minimizing bias introduced by sample overlap. Clinical evaluation of the prioritized candidates was subsequently conducted in an independently recruited Chinese sepsis cohort, accessible through the authors’ clinical practice, to examine whether the direction of genetically predicted regulation was reflected in observed gene expression. All analyses and reporting procedures followed the recommendations of the STROBE-MR guideline for Mendelian randomization studies (9).

### Clinical samples and ethical approval

Peripheral blood samples were collected from patients diagnosed with sepsis in the intensive care unit, and from healthy control individuals at the First Medical Center of the Chinese PLA General Hospital between November 1, 2022 and December 31, 2023. Sepsis was diagnosed according to the Sepsis-3 clinical definition, which requires confirmed or suspected infection accompanied by organ dysfunction defined as an increase of at least two points in the Sequential Organ Failure Assessment (SOFA) score relative to baseline. Individuals meeting these criteria during the recruitment period were eligible for inclusion. Patients were excluded if they were pregnant; had an immunosuppressed status, including long-term corticosteroid, immunosuppressant, antifungal, or antiviral therapy, HIV infection, other immunodeficiency, or a prior organ transplant; had received immune-enhancing agents such as thymosin, interleukins, or intravenous immunoglobulin; or presented with active malignancy. Healthy control individuals were confirmed to be free of active infection, inflammatory disease, and autoimmune disease, and had no chronic underlying conditions affecting the heart, brain, lungs, or kidneys. A total of twenty patients with sepsis and twenty healthy controls were included in the experimental validation cohort. Baseline characteristics of the validation cohort, including age, sex, SOFA score, and primary site of infection, are summarized in **Supplementary Table 1**. The study protocol was reviewed and approved by the Ethics Committee of the Chinese PLA General Hospital (approval No. S2022-735-01). Written informed consent was obtained from all participants or their legally authorized representatives prior to enrollment.

### GWAS datasets and molecular QTL resources

Summary statistics for sepsis were obtained from publicly available genome-wide association studies. The primary discovery analysis used the UK Biobank cohort, which included 11,643 individuals with sepsis and 474,841 controls of European ancestry. Sepsis cases were identified from linked hospital episode records using ICD-10 codes A02, A39, A40, and A41 in the primary or secondary diagnostic position, with self-reported cases and those recorded only in primary care excluded; the remaining cohort served as controls, and genetic associations were estimated with adjustment for age, sex, genotyping chip, and the first ten genetic principal components (15). Validation analyses were conducted using GWAS summary statistics from the FinnGen R12 release, including the six sepsis-related phenotypes available in this release: “Other septicemia”, “Streptococcal septicemia”, “Puerperal sepsis”, “Bacterial sepsis of newborn”, “Pneumococcal septicemia”, and “Hemophilus septicemia”. Like the UK Biobank, FinnGen is a population-based biobank of predominantly European ancestry in which sepsis cases are ascertained through linkage to nationwide hospital and health registry records using standardized ICD-coded diagnoses, providing a case-ascertainment strategy broadly comparable to that of the discovery dataset. Among these, “Other septicemia” (ICD-10 A41) was designated as the primary validation phenotype. Together with “Streptococcal septicemia” (ICD-10 A40), it is one of two FinnGen phenotypes that correspond directly to a component of the discovery phenotype from UK Biobank. It was selected as primary because it spans multiple bacterial etiologies rather than a single pathogen, better reflecting the etiological breadth of the discovery cohort. However, it does not capture the Salmonella- or meningococcus-associated cases (ICD-10 A02, A39) included in the discovery phenotype, for which FinnGen provides no corresponding endpoint. “Streptococcal septicemia” and the remaining, more narrowly defined FinnGen R12 phenotypes were used for subtype-specific validation, testing whether the identified associations held across clinically distinct presentations of sepsis. Detailed information regarding all outcome datasets is provided in **Supplementary Table 2**.

A comprehensive list of mitochondria-related genes was curated from the MitoCarta 3.0 database (**Supplementary Table 3**), which systematically catalogs human mitochondrial proteins and their associated genes based on experimental evidence and integrative annotations (16). In total, 1,136 mitochondrial genes were included in the present analysis. Molecular QTL data spanning four layers of gene regulation were incorporated into the analysis: DNA methylation, whole-blood and single-cell gene expression, and circulating protein abundance. These layers traced genetically mediated effects from an upstream epigenetic mark to the downstream protein product. Genetic regulation of gene expression in whole blood was obtained from the eQTLGen consortium, which analyzed expression quantitative trait loci across 31,684 individuals from 37 independent cohorts, with cohort-specific adjustment for age, sex, genotype principal components, and PEER factors (17). To investigate immune cell-specific regulatory effects, single-cell expression quantitative trait locus (sc-eQTL) data were obtained from the OneK1K cohort, comprising single-cell RNA sequencing profiles of 1.27 million peripheral blood mononuclear cells across 14 immune cell subsets from 982 donors, with adjustment for PEER factors, sex, and genotype principal components within each cell type (14). DNA methylation quantitative trait loci were obtained from a meta-analysis of whole-blood DNA methylation, profiled with the Illumina HumanMethylation450 array, combining the Brisbane Systems Genetics Study and the Lothian Birth Cohorts, adjusted for age, sex, and estimated blood cell-type proportions (18). Genetic associations with circulating protein abundance were obtained from a SomaScan-based plasma proteomic QTL study including 35,559 Icelanders, adjusted for age, sex, genotype principal components, and sample collection batch (19). Because all genetic association datasets used in this study were obtained from previously published resources with existing ethical approval, no additional ethical approval was required for the use of these summary statistics.

### Summary-data-based Mendelian randomization analysis

The SMR method (SMR software, version 1.4.0) was applied to integrate GWAS summary statistics with molecular QTL data to test whether genetic variants associated with molecular traits also influence disease risk through the same regulatory pathway (10). In the primary analysis, molecular QTLs, comprising eQTLs, mQTLs, sc-eQTLs, and pQTLs, served as the exposure, and sepsis GWAS summary statistics served as the outcome. In this framework, a single top cis-acting QTL variant for each molecular trait was selected as the instrumental variable in the original SMR model. Cis-QTLs were defined as variants located within ±1,000 kilobases of the gene locus, with a minor allele frequency greater than 0.01, and reaching genome-wide significance (P < 5 × 10^-8^) (20). Variants showing allele frequency discrepancies greater than 0.1 between datasets were excluded to minimize potential bias arising from allele mismatching across datasets, a filter that also indirectly controls for ambiguous palindromic SNPs. Allele harmonization between exposure and outcome datasets was performed using the SMR software’s built-in allele-matching procedure. All analyses were conducted on the GRCh37/hg19 genome build; pQTL summary statistics, originally reported in GRCh38, were lifted over to GRCh37 prior to analysis. Linkage disequilibrium among variants within each cis-regulatory region was estimated using the 1000 Genomes Project Phase 3 European reference panel (21), consistent with the predominantly European ancestry of the GWAS and QTL datasets used in this study. Instrument strength was assessed using the F-statistic, calculated as the squared ratio of the SNP-exposure effect estimate to its standard error, with F > 10 conventionally indicating a low likelihood of weak-instrument bias.

In addition to the original SMR model, the SMR-multi extension was applied to account for the possibility that multiple causal variants may exist within a cis-regulatory region, thereby reducing potential bias associated with the assumption of a single instrumental variant (11). Beyond evaluating associations between molecular traits and sepsis risk, additional SMR analyses were conducted to investigate potential regulatory cascades across biological layers, including analyses in which mQTLs were treated as exposures and eQTLs as outcomes.

To reduce the likelihood of spurious findings caused by linkage disequilibrium, the heterogeneity in dependent instruments (HEIDI) test was performed as part of the SMR procedure. The HEIDI test evaluates whether the observed association pattern across variants is consistent with a shared causal variant. Associations with a HEIDI P value below 0.01 were considered likely to be driven by linkage rather than pleiotropy and were therefore excluded from further interpretation. Candidate loci were required to satisfy three criteria simultaneously: a nominal SMR significance threshold (*P*_SMR_ < 0.05), statistical support from the multi-variant SMR model (*P*_SMR-multi_ < 0.05), and absence of evidence for linkage according to the HEIDI test (*P*_HEIDI_ > 0.01). These loci were subsequently evaluated using colocalization analysis.

### Colocalization analysis

To further determine whether the molecular QTL signals and sepsis GWAS associations observed within a genomic region were attributable to the same causal variant, Bayesian colocalization analysis was performed. The analysis was conducted using the R package coloc, which estimates posterior probabilities for five alternative hypotheses (H0-H4) describing the relationship between two association signals (22, 23). These hypotheses correspond to scenarios in which no causal variants are present for either trait (H0), a causal variant exists only for the molecular trait (H1), a causal variant exists only for the disease trait (H2), two independent causal variants affect each trait separately (H3), or a single shared causal variant influences both traits (H4). All single nucleotide polymorphisms located within a ±1,000 kb window around the lead cis-QTL were included in the analysis. Evidence supporting colocalization was considered suggestive when the posterior probability for H4 (PPH4) exceeded 0.5, indicating that both traits were likely influenced by the same underlying genetic variant (24–26). A PPH4 value above 0.5 indicates that a shared causal variant is the relatively most probable explanation among the five hypotheses defined by Giambartolomei et al. (22), reflecting moderate rather than strong colocalization evidence. In the present study, it was treated only as a complementary indicator of colocalization support rather than an independent gating criterion for candidate gene prioritization (24, 27) Candidate gene prioritization in the present study was based on the SMR and HEIDI criteria described above; PPH4 was applied as a complementary indicator of colocalization support among these prioritized candidates, reflecting the relative strength of evidence for a shared causal variant rather than serving as an independent gating criterion.

### RNA extraction and reverse transcription

Total RNA was isolated from peripheral whole blood samples using TRIzol reagent (Life Technologies, 15596-026) following the manufacturer’s instructions. After phase separation with chloroform, RNA was precipitated from the aqueous phase using isopropanol. The RNA pellet was subsequently washed with ethanol, air-dried, and resuspended in nuclease-free water. RNA concentration and purity were measured using a NanoDrop spectrophotometer (Thermo Fisher Scientific). Complementary DNA was synthesized using reverse transcription kits from Servicebio (G3337-50) and TOYOBO (FSQ-301) according to the manufacturers’ protocols.

### Quantitative real-time PCR validation

Quantitative real-time PCR experiments were performed using the Bio-Rad CFX96 Touch Real-Time PCR Detection System. Reactions were carried out in a final volume of 20 μL using SYBR Green reagents (Servicebio G3326 or TOYOBO QPX-201) following the manufacturers’ instructions. All reactions were performed in technical triplicate, with three replicate wells per sample and target gene. β-actin (ACTB) was used as the internal reference gene to normalize gene expression levels, selected according to the predefined experimental protocol as a housekeeping gene commonly applied in RT-qPCR analysis of peripheral blood samples (28). Candidate mitochondrial genes prioritized through SMR analyses were selected for experimental validation, including *AFG1L*, *AK4*, *COX4I1*, *MICOS10*, *MPC2*, *MTFMT*, *NDUFB5*, *NDUFV3*, *PHB2*, *PPOX*, *SURF1*, and *TUFM*. The gene *SLC25A35* could not be reliably detected under the experimental conditions used in this study. Primer sequences for all target genes are provided in **Supplementary Table 4**. For each target gene and the reference gene, the Ct value used for calculation was the mean of three technical replicate wells; replicate measurements showing technical inconsistency were excluded during routine quality control. Relative gene expression levels were calculated using the 2^-ΔΔCt^ method. For each sample, ΔCt was obtained by subtracting the Ct value of the reference gene (ACTB) from that of the target gene; ΔΔCt was then calculated as the difference between the ΔCt of each sample and the mean ΔCt of the healthy control group, and relative expression was expressed as 2^-ΔΔCt^.

### Results visualization and statistical analysis

All statistical analyses were conducted using R software (version 4.3.0). Visualization of genome-wide results was performed using the CMplot package for Manhattan plots and the forestplot package for forest plot generation. Visualization scripts for SMRLocusPlot and SMREffectPlot were adapted from the original SMR publication (10). Because multiple molecular traits were evaluated across several analytical layers, multiple testing was controlled using the Benjamini-Hochberg false discovery rate (FDR) procedure. Relative expression values (2^-ΔΔCt^) for the qPCR validation data were tested for normality within each group using the Shapiro-Wilk test, and seven of the twelve genes showed a non-normal distribution in at least one group. Group comparisons of relative expression values (2^-ΔΔCt^) for all twelve genes were therefore performed uniformly using the Mann-Whitney U test, irrespective of individual gene-level normality, and multiple testing across genes was controlled using the Benjamini-Hochberg procedure. Associations satisfying the predefined SMR screening criteria were reported as prioritized candidates for further investigation, while FDR-adjusted results were provided to assist interpretation of statistical robustness. A consolidated summary of the key analytical parameters described throughout the Methods, including software versions, genome build, LD reference panel, and instrument and colocalization thresholds, is provided in **Supplementary Table 5**.

## Results

### Mitochondrial gene expression signals associated with sepsis risk

**Figure 2:**
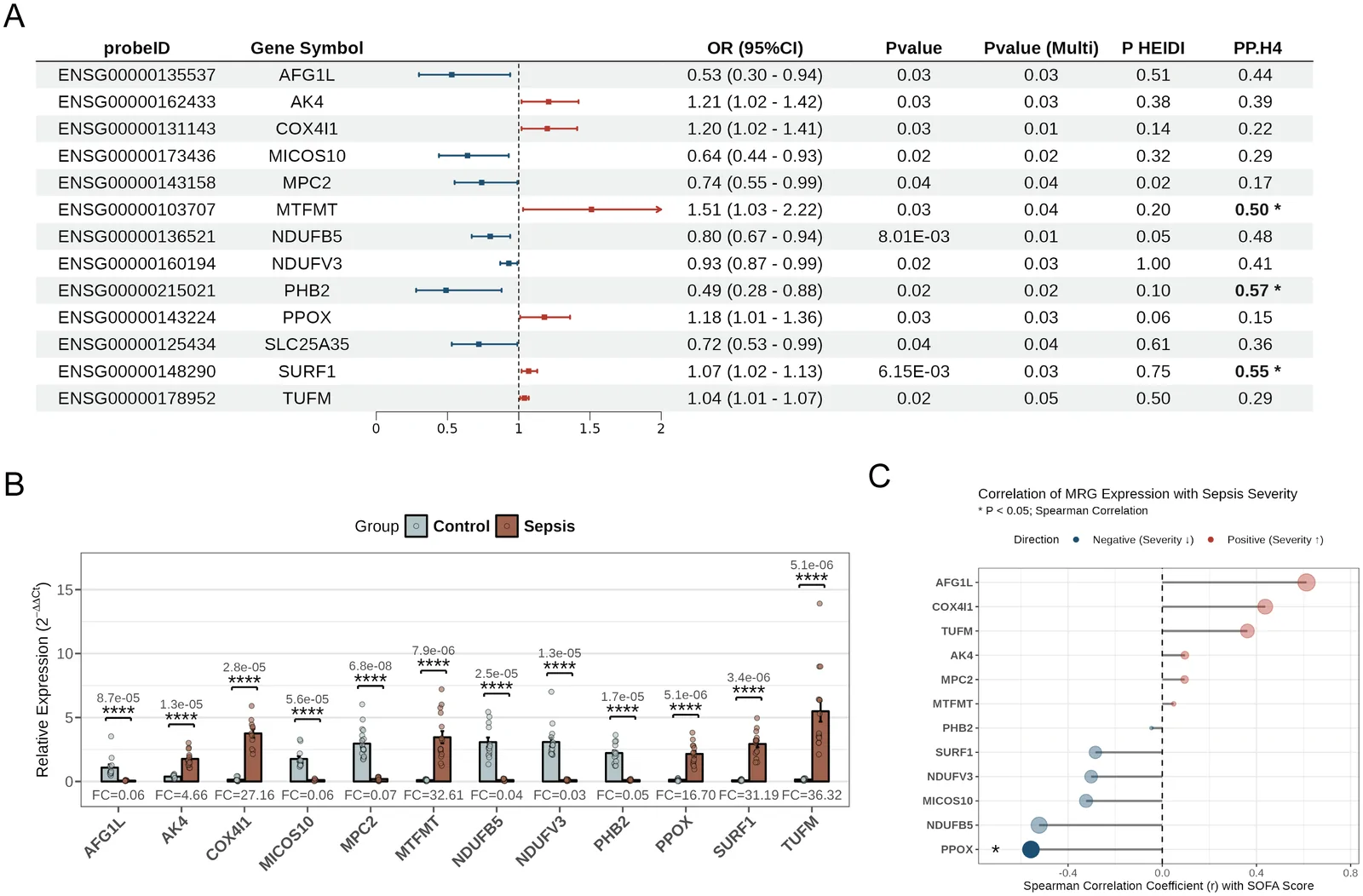
Blood eQTL-based SMR analysis and clinical validation of prioritized mitochondria-related genes. **(A)** Forest plot showing the associations between genetically predicted expression of 13 prioritized mitochondria-related genes and sepsis risk based on blood eQTL data. Odds ratios (ORs) and 95% confidence intervals (CIs) are displayed. Asterisks (*) indicate loci with suggestive evidence of colocalization (PPH4 > 0.5). **(B)** Relative mRNA expression levels of prioritized genes in peripheral blood samples from septic patients (n = 20) and healthy controls (n = 20), measured by quantitative real-time PCR (qPCR). Exact fold change and P values for each gene are annotated above the corresponding bars. Data are presented as mean ± standard deviation (SD). Group comparisons were performed using the Mann-Whitney U test. ***P < 0.0001. **(C)** Spearman correlation analysis between the relative expression levels of prioritized genes and Sequential Organ Failure Assessment (SOFA) scores in septic patients. Colors indicate the direction of correlation (blue for positive and red for negative correlations). *P < 0.05.

Integration of blood eQTL data with sepsis GWAS summary statistics identified multiple mitochondria-related genes whose genetically predicted expression showed evidence of association with sepsis susceptibility. Using the predefined screening criteria (*P*_SMR_ < 0.05, *P*_SMR-multi_ < 0.05, and *P*_HEIDI_ > 0.01), thirteen genes passed the SMR filtering procedure (**Figure 2A**; **Supplementary Table 6**). None of these thirteen genes showed evidence of heterogeneity or pleiotropy attributable to linkage disequilibrium, with *P*_HEIDI_ values above the prespecified threshold for every locus (**Supplementary Table 6**). Instrument strength was strong across all thirteen prioritized genes, with F-statistics ranging from 40.78 to 16,469.44, exceeding the conventional threshold for a strong instrument (**Supplementary Table 7**). Among these candidates, six genes showed positive associations between genetically predicted expression and sepsis risk. Increased expression of *AK4* (OR = 1.21, 95% CI 1.02-1.42; *P*_SMR_ = 0.026; *P*_HEIDI_ = 0.379), *COX4I1* (OR = 1.20, 95% CI 1.02-1.41; *P*_SMR_ = 0.031; *P*_HEIDI_ = 0.137), *MTFMT* (OR = 1.51, 95% CI 1.03-2.22; *P*_SMR_ = 0.035; *P*_HEIDI_ = 0.199), *PPOX* (OR = 1.18, 95% CI 1.01-1.36; *P*_SMR_ = 0.034; *P*_HEIDI_ = 0.059), *SURF1* (OR = 1.07, 95% CI 1.02-1.13; *P*_SMR_ = 0.006; *P*_HEIDI_ = 0.747), and *TUFM* (OR = 1.04, 95% CI 1.01-1.07; *P*_SMR_ = 0.023; *P*_HEIDI_ = 0.498) was associated with higher sepsis risk. Other prioritized genes showed inverse or modest associations (**Supplementary Table 6**). A genome-wide overview of the lead cis-eQTL signals across all thirteen prioritized genes is provided in **Supplementary Figure 1**.

Bayesian colocalization analysis provided additional insight into the genetic architecture underlying these signals. Three loci showed suggestive evidence of a shared causal variant between eQTL and sepsis GWAS signals (PPH4 > 0.5), whereas several genes displayed weaker support for colocalization (**Figure 2A**). For example, the *AK4* locus demonstrated moderate colocalization probability (PPH4 = 0.39), suggesting that the observed SMR signal may partly reflect nearby linkage structure rather than a single shared variant.

Additional analyses were conducted using sepsis-related GWAS phenotypes from the FinnGen R12 release. The phenotype “Other septicemia” was treated as the primary validation dataset. In this analysis, genetically predicted expression of *MTFMT* showed a significant association with sepsis susceptibility (OR = 1.26, 95% CI 1.04-1.54; *P*_SMR_ = 0.018; *P*_HEIDI_ = 0.319), consistent with the direction of effect observed in the discovery analysis (**Supplementary Table 8**). Subtype-specific analyses were further performed using additional FinnGen phenotypes, including “Streptococcal septicemia”, “Puerperal sepsis”, “Bacterial sepsis of newborn”, “Pneumococcal septicemia”, and “Hemophilus septicemia”. Among these, a significant association was observed for *SLC25A35* in the Bacterial sepsis of newborn cohort (OR = 0.18, 95% CI 0.04-0.86; *P*_SMR_ = 0.032; *P*_HEIDI_ = 0.174), whereas no significant signals were detected in the remaining subtype analyses (**Supplementary Table 8**). These results provide additional context for interpreting the mitochondrial candidate genes identified in the discovery stage.

### Expression patterns of prioritized genes in clinical sepsis samples

Clinical characteristics of the qPCR validation cohort are provided in **Supplementary Table 1**. Expression levels of the SMR-prioritized genes were next examined in peripheral blood samples from patients with sepsis and healthy controls using quantitative PCR. Twelve genes were successfully quantified in clinical samples, whereas *SLC25A35* was not detectable under the experimental conditions used in this study. Effect sizes for the observed group differences were large across all twelve genes (Cohen’s d exceeding 1.5 in eleven of twelve genes), and *post hoc* statistical power was adequate (≥0.80) for eleven of the twelve genes tested (**Supplementary Table 9**). ACTB Ct values did not show an apparent systematic difference between the sepsis and control groups after technical quality control.

Comparison of gene expression levels showed differential expression for the quantified candidate genes between sepsis patients and controls (**Figure 2B**; **Supplementary Table 9**). Although the magnitude of expression change varied across genes, these observations indicate that several genetically prioritized candidates also show transcriptional alterations in clinical samples.

Associations between gene expression and disease severity were further evaluated using Spearman correlation analysis with SOFA scores. Among the tested genes, *PPOX* expression showed a significant negative correlation with SOFA score (P < 0.05), indicating that lower *PPOX* expression was associated with greater organ dysfunction (**Figure 2C**). Other genes showed heterogeneous correlation patterns that did not reach statistical significance. Notably, the direction of expression change for several genes, including *AK4*, was consistent with the direction of effect predicted by the genetic analysis, suggesting concordance between genetically predicted regulation and observed transcriptional patterns in sepsis.

### Immune cell-specific regulatory signals identified from single-cell eQTL data

**Figure 3:**
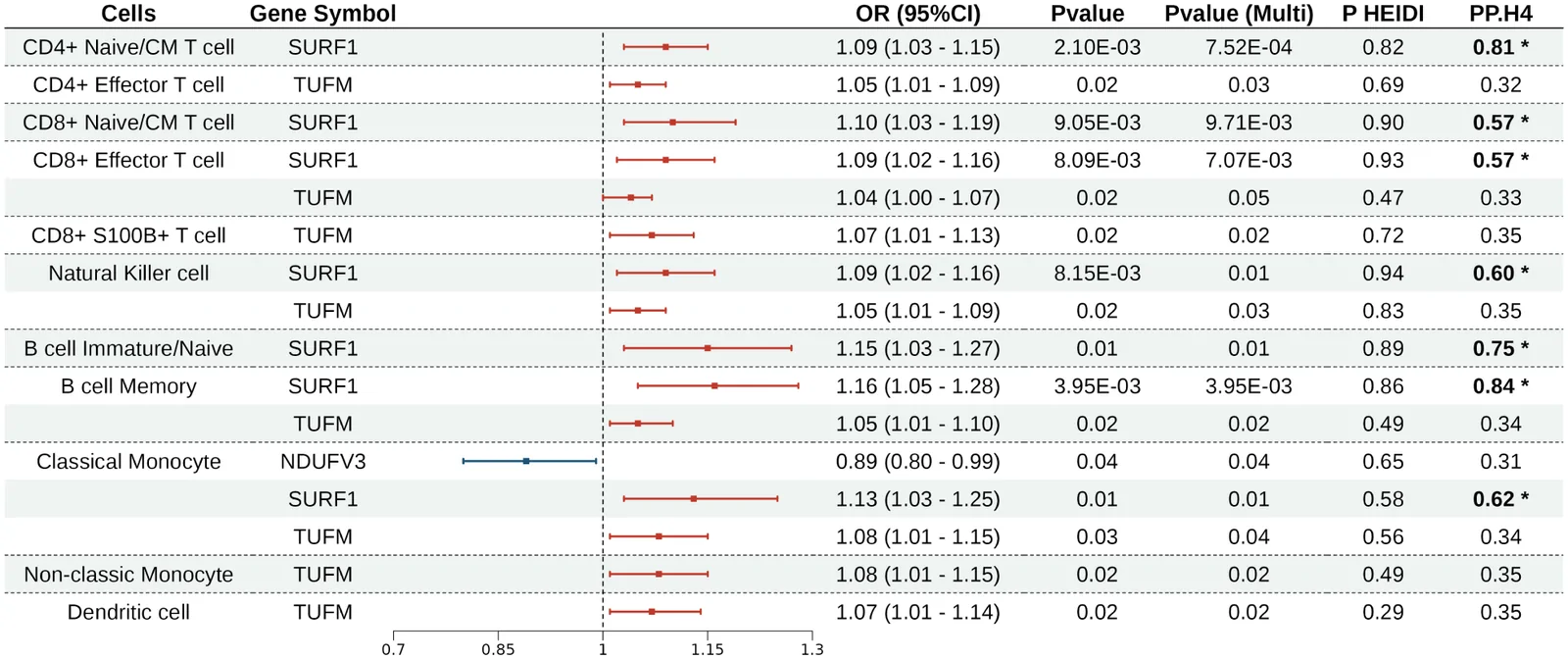
Immune cell–specific regulatory signals of prioritized genes revealed by single-cell eQTL analysis. Forest plot summarizing cell-type–specific SMR associations and colocalization probabilities for prioritized mitochondrial genes across multiple immune cell subpopulations in the OneK1K cohort. The results indicate that genetic regulation of certain targets, such as *SURF1*, is enriched within specific adaptive immune compartments (e.g., memory B cells and naive/central memory T cells). Asterisks (*) denote suggestive evidence of a shared causal variant between single-cell eQTL and sepsis GWAS signals (PPH4 > 0.5).

Single-cell eQTL analyses based on immune cell populations from the OneK1K cohort provided further insight into the cellular context of the identified transcriptional associations. Several mitochondrial genes showed evidence of cell-type-specific regulatory effects across immune cell subsets (**Figure 3**; **Supplementary Table 10**).

The gene *SURF1* displayed consistent association signals across multiple lymphocyte populations, including CD4^+^ naïve or central memory T cells, CD8^+^ T cell subsets, natural killer cells, and B cell populations. The strongest colocalization evidence for *SURF1* was observed in memory B cells (PPH4 = 0.84) and CD4^+^ naïve or central memory T cells (PPH4 = 0.81). *TUFM* also demonstrated associations across several immune cell types, although with weaker colocalization support. In addition, *NDUFV3* showed an association signal within classical monocytes. These findings suggest that mitochondrial gene regulation associated with sepsis susceptibility may occur within specific immune cell populations rather than uniformly across all circulating cells.

### Epigenetic signals reveal regulatory mechanisms linking DNA methylation to gene expression

**Figure 4:**
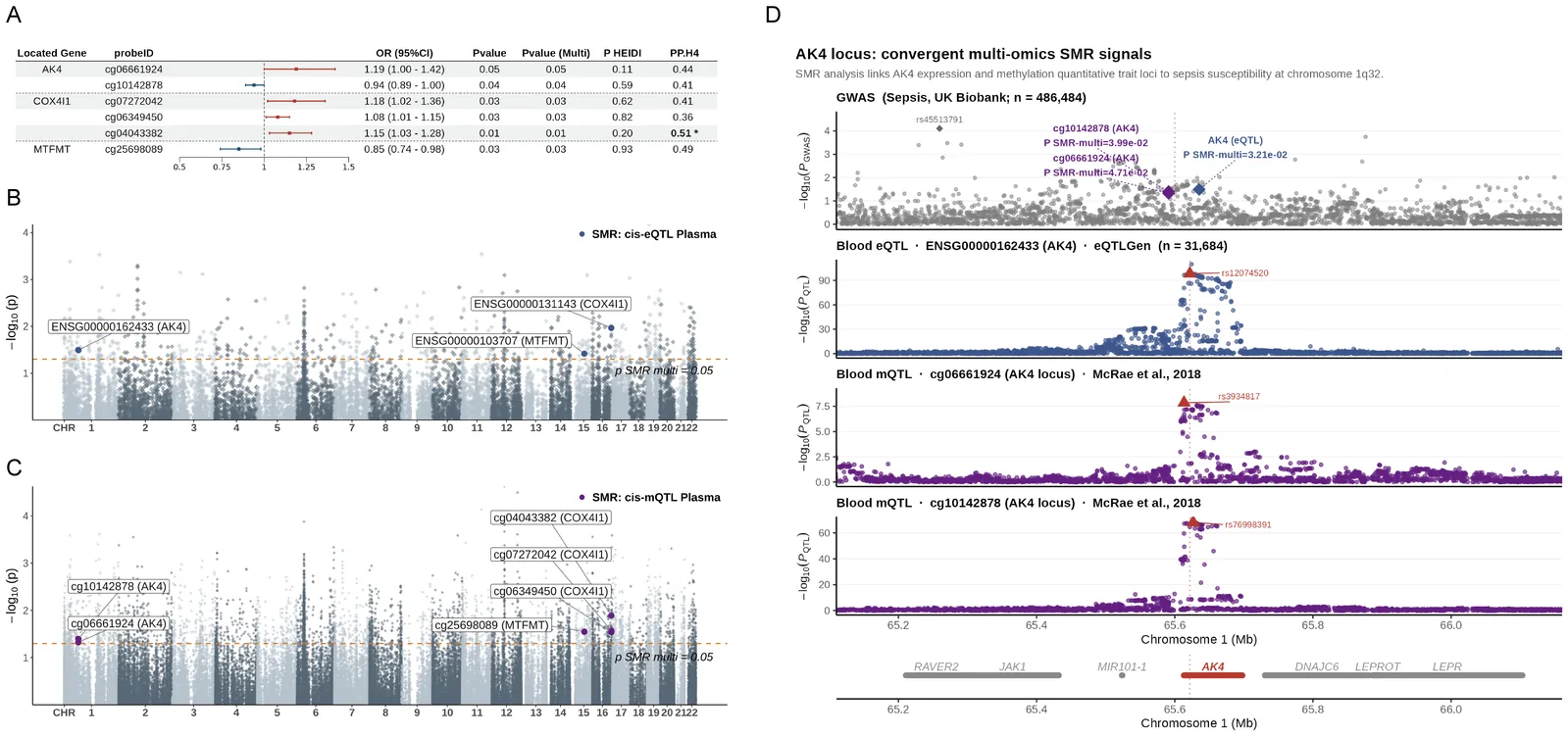
Epigenetic regulatory signals linking DNA methylation to gene expression. **(A)** Forest plot showing SMR associations between genetically predicted DNA methylation levels (mQTLs) and sepsis risk. Only CpG sites annotated to genes prioritized in the eQTL analysis (*AK4*, *COX4I1*, and *MTFMT*) are displayed. **(B)** Manhattan plot of the eQTL-based SMR analysis for sepsis. Highlighted genes correspond to those showing convergent signals with the epigenetic analysis. The dashed horizontal line indicates the significance threshold (*P*_SMR-multi_ = 0.05). **(C)** Manhattan plot of the mQTL-based SMR analysis for sepsis. Highlighted points represent CpG sites annotated to prioritized eQTL genes, suggesting a potential multi-omics regulatory cascade. **(D)** Regional association plot across the *AK4* locus, showing sepsis GWAS, blood eQTL, and blood mQTL (cg06661924 and cg10142878) signals in the same genomic window. The lead cis-QTL variant used as the instrumental variable in each analysis is indicated (eQTL: rs12074520; mQTL: rs3934817 and rs76998391, respectively). Gene annotations for *AK4* and flanking genes are shown at the bottom.

Additional analyses were performed to investigate potential upstream epigenetic regulation by integrating methylation QTL (mQTL) data with sepsis GWAS summary statistics. Multiple CpG sites within mitochondria-related gene regions showed associations between genetically predicted methylation levels and sepsis susceptibility (**Supplementary Figure 2**; **Supplementary Table 11**). Within the *AK4* locus, methylation at cg06661924 was positively associated with susceptibility (OR = 1.19, 95% CI 1.00-1.42; *P*_SMR_ = 0.047; *P*_HEIDI_ = 0.113), whereas a nearby CpG site, cg10142878, showed a modest inverse association (OR = 0.94, 95% CI 0.89-1.00; *P*_SMR_ = 0.035; *P*_HEIDI_ = 0.588). Additional CpG sites within genes including *COX4I1* and *MTFMT* also satisfied the predefined SMR screening criteria (**Figures 4A–C**; **Supplementary Table 11**). Bayesian colocalization analysis identified multiple methylation loci with evidence of shared causal variants with sepsis risk (PPH4 > 0.5), including loci annotated to *ISCA2*, *MRPL44*, and *SHMT2* (**Supplementary Figure 2**).

Additional analyses were then performed using sepsis-related phenotypes from the FinnGen R12 release. In the primary validation phenotype, other septicemia, three CpG sites annotated to *VARS2* remained significant after false discovery rate correction, with consistent positive effect estimates (cg07945879: OR = 1.24, 95% CI 1.10-1.39, *P*_SMR_ = 2.64×10^-4^, *P*_HEIDI_ = 0.184; cg26467571: OR = 1.23, 95% CI 1.10-1.37, *P*_SMR_ = 3.22×10^-4^, *P*_HEIDI_ = 0.478; and cg02601318: OR = 1.25, 95% CI 1.11-1.41, *P*_SMR_ = 1.92×10^-4^, *P*_HEIDI_ = 0.795). In addition, cg20917322 within *CCDC51* and cg07884270 within *TRMT1* showed nominal associations with the same phenotype (**Supplementary Table 12**). Subtype-specific analyses revealed heterogeneous methylation signals across clinically distinct sepsis phenotypes. For example, cg24429974 annotated to *TOMM40L* was positively associated with puerperal sepsis (OR = 1.13, 95% CI 1.03-1.24; *P*_SMR_ = 0.010, *P*_HEIDI_ = 0.147) but inversely associated with bacterial sepsis of newborn (OR = 0.58, 95% CI 0.36-0.94; *P*_SMR_ = 0.029, *P*_HEIDI_ = 0.817). A significant association with pneumococcal septicemia was observed for cg01420215 (OR = 0.74, 95% CI 0.57-0.95; *P*_SMR_ = 0.020, *P*_HEIDI_ = 0.975), annotated to MRPL44, consistent with the same CpG site supported by colocalization evidence with sepsis risk in the discovery-stage analysis (OR = 0.88, 95% CI 0.81 - 0.96; *P*_SMR_ = 0.003, *P*_HEIDI_ = 0.406, PPH4 = 0.778), while cg16340446, annotated to MRPL47, showed a significant association with Hemophilus septicemia (OR = 0.11, 95% CI 0.01-0.94; *P*S_MR_ = 0.043, *P*_HEIDI_ = 0.074; **Supplementary Table 12**).

Among the loci supported across both methylation and expression layers, *AK4* was the only gene showing convergent evidence in both analyses (**Supplementary Figure 3**). Genetically predicted *AK4* expression was positively associated with sepsis risk (OR = 1.21, 95% CI 1.02-1.42; *P*_SMR_ = 0.026; *P*_HEIDI_ = 0.379), while methylation at cg06661924 showed a concordant association with susceptibility (OR = 1.19, 95% CI 1.00-1.42; *P*_SMR_ = 0.047; *P*_HEIDI_ = 0.113). Secondary SMR analyses using mQTLs as exposures and eQTLs as outcomes identified a strong association between cg06661924 methylation and AK4 expression (β = 0.89, 95% CI 0.57-1.21; *P*_SMR_ = 5.33 × 10^-8^; *P*_HEIDI_ = 0.030; **Supplementary Table 13**). A regional overview of the GWAS, eQTL, and mQTL association signals across the AK4 locus is provided in **Figure 4D**.

### analyse Protein-level s reveal limited downstream associations

Protein-level associations were evaluated by integrating pQTL data with sepsis GWAS summary statistics. Compared with the transcriptomic and epigenetic layers, fewer associations were detected at the protein level (**Supplementary Table 14**).

Only DUT satisfied the predefined SMR screening criteria in the discovery analysis (*P*_SMR_ = 0.039; *P*_HEIDI_ = 0.922), with higher genetically predicted protein abundance associated with increased sepsis risk (OR = 1.67, 95% CI 1.03–2.72; **Supplementary Figure 4**).

Subtype-specific analyses also identified significant associations for DUT in the FinnGen R12 streptococcal septicemia cohort (OR = 2.46, 95% CI 1.01-5.98; *P*_SMR_ = 0.047; *P*_HEIDI_ = 0.310) and the pneumococcal septicemia cohort (OR = 6.40, 95% CI 1.42-28.77; *P*_SMR_ = 0.016; *P*_HEIDI_ = 0.129), in the same direction as the discovery analysis (**Supplementary Table 15**).

### Integrated multi-omics evidence of prioritized genes

**Figure 5:**
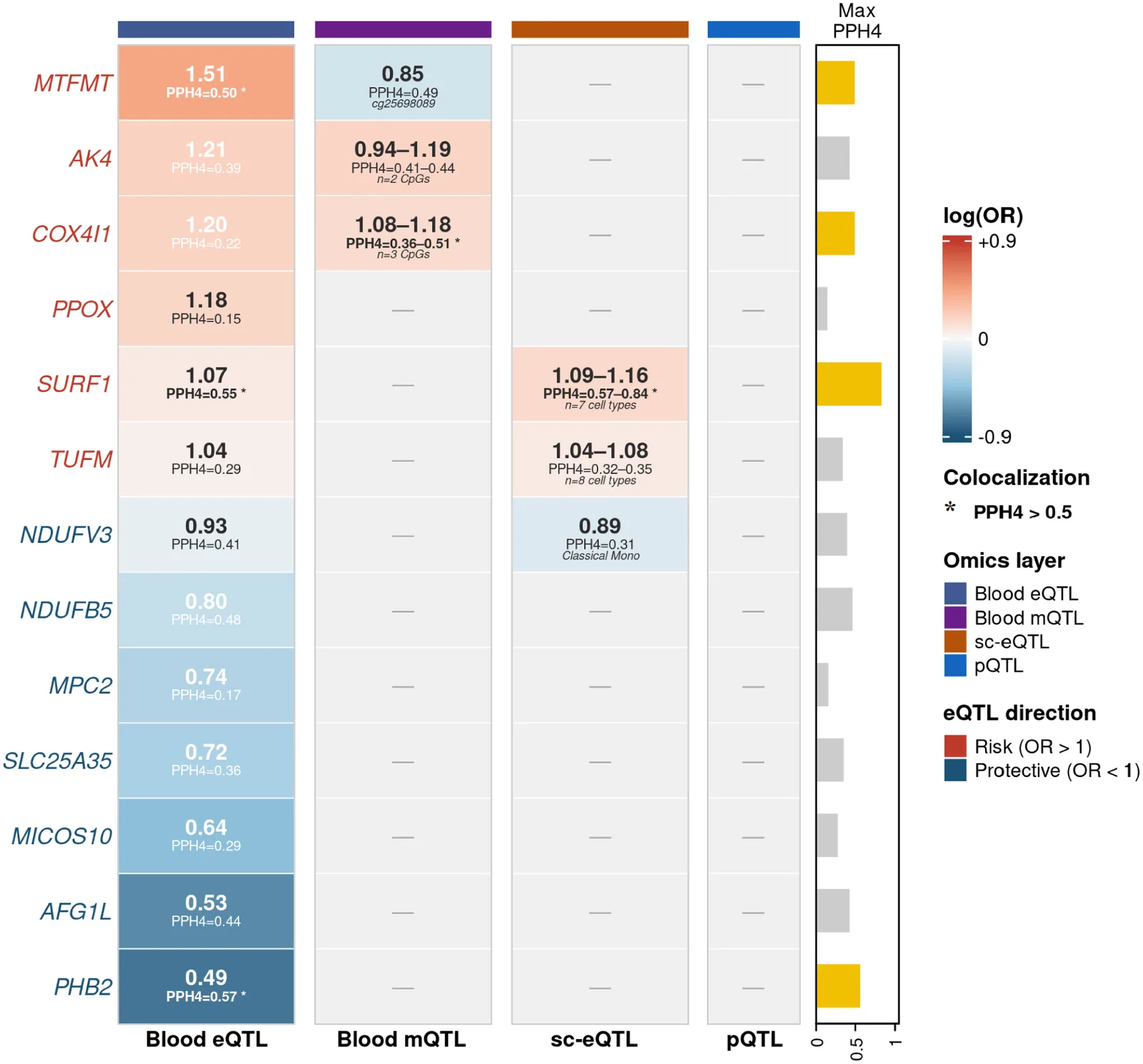
Multi-omics evidence matrix for prioritized mitochondria-related genes. Heatmap summarizing SMR effect estimates (odds ratio) for the thirteen prioritized genes across four molecular layers: blood eQTL, blood mQTL, single-cell eQTL (sc-eQTL), and pQTL. Cell values indicate the odds ratio for each gene-layer combination with an available SMR signal. Where a gene showed more than one significant result within a layer, such as multiple CpG sites for blood mQTL or multiple immune cell types for sc-eQTL, the range across all such results is shown instead, along with the number of underlying results. Single-result cells instead show the specific CpG probe or cell-type label. Gray cells indicate no signal meeting the SMR screening criteria in that layer. PPH4 values are shown in bold with an asterisk where the maximum PPH4 for that cell exceeds 0.5, denoting suggestive colocalization support. The adjacent bar chart shows the maximum PPH4 value observed across all layers for each gene, with gold bars indicating PPH4 > 0.5. Gene labels are colored by the direction of the blood eQTL association with sepsis risk (red, odds ratio > 1; blue, odds ratio < 1).

An integrated summary of the evidence across all four molecular layers, blood eQTL, blood mQTL, single-cell eQTL, and pQTL, is presented for the thirteen prioritized genes in **Figure 5** and **Supplementary Table 16**. Four of these genes, MTFMT, COX4I1, SURF1, and PHB2, showed at least one molecular layer with a posterior probability of colocalization (PPH4) exceeding 0.5, with SURF1 showing the strongest support (PPH4 = 0.84 in memory B cells). AK4 showed converging evidence from both the eQTL and mQTL layers, despite moderate colocalization probabilities at either individual layer (PPH4 = 0.39 and 0.44, respectively).

## Discussion

In this study, we used a multi-omics SMR framework to evaluate whether mitochondria-related molecular traits are genetically linked to sepsis susceptibility. By integrating blood-based eQTL, mQTL, single-cell eQTL, and pQTL data with sepsis GWAS summary statistics, we identified a set of prioritized mitochondrial candidates that may be relevant to sepsis risk. The overall pattern that emerged was not one of broad and consistent associations across all molecular layers. Instead, only a limited number of genes showed convergent evidence across complementary analyses. Among these candidates, *AK4* was notable because it showed signals at both the methylation and transcriptional levels, and the genetically predicted direction of effect was consistent with the increased expression observed in peripheral blood from septic patients. In parallel, the single-cell eQTL analysis highlighted *SURF1* as a gene with relatively consistent immune cell-specific regulatory signals, suggesting that part of the transcriptional association detected in whole blood may originate from particular immune cell populations rather than from a uniform effect across circulating cells.

Among the prioritized genes, *AK4* deserves particular attention because it was the only candidate showing convergent evidence across the mQTL-to-sepsis, eQTL-to-sepsis, and mQTL-to-eQTL analyses. *AK4* encodes a mitochondrial adenylate kinase that participates in adenine nucleotide homeostasis and contributes to the maintenance of mitochondrial ATP balance (29). This role is biologically relevant in sepsis, a condition characterized by substantial metabolic stress and mitochondrial dysfunction (7, 30). Although direct human genetic evidence linking *AK4* to sepsis has been limited, experimental studies provide supporting biological context. In macrophages, *AK4* has been shown to promote inflammatory gene expression through mechanisms involving HIF1α signaling and inhibition of AMP-activated protein kinase (AMPK), thereby enhancing glycolytic metabolism and inflammatory activation (31). Suppression of *AK4* expression reduced glycolytic activity and attenuated inflammatory responses in macrophages (31). These observations suggest that *AK4* may contribute to inflammation-associated metabolic reprogramming. Consistent with this possibility, our data showed that genetically predicted *AK4* up-regulation was associated with increased sepsis risk, while methylation at cg06661924 exhibited a concordant association with susceptibility. Secondary SMR analysis further indicated that this CpG site may influence *AK4* expression. The qPCR results provided additional support at the clinical level, showing elevated *AK4* expression in peripheral blood from septic patients. Taken together, these findings support *AK4* as a prioritized candidate for further functional investigation, particularly in the context of inflammation-related metabolic adaptation.

The immune cell-specific eQTL analysis provided additional insight into the cellular context of the observed associations. *SURF1* showed repeated regulatory signals across several immune cell subsets, with relatively strong colocalization evidence in memory B cells and naïve or central-memory T-cell populations. This pattern suggests that the transcriptional relationship between *SURF1* and sepsis risk may be concentrated within selected immune compartments rather than broadly distributed across all leukocyte populations. *SURF1* encodes a mitochondrial protein required for the proper assembly of cytochrome c oxidase (complex IV) of the respiratory chain, and pathogenic variants in this gene are known to cause severe mitochondrial respiratory chain deficiency (32, 33). Because mitochondrial metabolism plays a central role in regulating immune cell activation and inflammatory responses, including immune cell death pathways such as NETosis and pyroptosis that shape the host response during sepsis (34–36), altered regulation of *SURF1* could plausibly influence host responses during infection. Although direct evidence linking *SURF1* to sepsis remains limited, the cell-specific regulatory pattern observed in this study suggests that it may represent a promising target for follow-up mechanistic investigation.

The findings for *SLC25A35* were more limited and should be interpreted cautiously. At the eQTL level, genetically predicted higher expression of *SLC25A35* was associated with reduced sepsis risk. *SLC25A35* belongs to the SLC25 family of mitochondrial carrier proteins, which facilitate metabolite exchange across the mitochondrial inner membrane and therefore play important roles in maintaining mitochondrial metabolic homeostasis (37). Despite this functional relevance, the evidence for *SLC25A35* in the present analysis was less consistent than that observed for AK4 or SURF1. The colocalization support was modest, and SLC25A35 expression remained below the detection threshold in the qPCR assays under the experimental conditions used, and supportive signals were mainly observed in subtype analyses rather than consistently across all datasets. Therefore, *SLC25A35* should be regarded as a suggestive protective candidate rather than a strongly supported gene. Future investigations using larger datasets or functional assays may help clarify whether mitochondrial transport processes involving *SLC25A35* influence host responses during sepsis.

Beyond the genes highlighted by genetic prioritization, the clinical validation data also revealed additional observations of potential biological interest. In particular, expression of *PPOX* in peripheral blood showed a negative correlation with the SOFA score in septic patients, indicating that lower *PPOX* expression was associated with more severe organ dysfunction. This finding stands alongside the eQTL-based result described above, in which genetically predicted higher PPOX expression was associated with increased sepsis risk. These two associations describe different clinical outcome dimensions: susceptibility to developing sepsis, as captured by the eQTL-based analysis, and severity of organ dysfunction once sepsis has occurred, as captured by the SOFA correlation. A genetic variant that shapes the likelihood of crossing into disease need not bear the same relationship to the degree of organ dysfunction that follows, and the two associations are therefore not in direct conflict. *PPOX* encodes protoporphyrinogen oxidase, a mitochondrial enzyme involved in heme biosynthesis. Because mitochondrial respiratory function depends on heme-containing complexes within the electron transport chain (38), alterations in *PPOX* expression may reflect changes in mitochondrial metabolic capacity during systemic inflammation and infection-related stress (39). Although this observation does not establish a causal relationship, it suggests that mitochondrial metabolic pathways associated with heme synthesis may be linked to disease severity in sepsis and therefore merit further investigation.

The methylation analyses provided a broader view of the upstream regulatory landscape. Numerous CpG sites satisfied the predefined SMR screening criteria, but only a subset demonstrated stronger colocalization support. Overall, the methylation signals appeared more heterogeneous than the transcriptional signals, suggesting that epigenetic regulation of sepsis susceptibility may involve multiple locus-specific mechanisms rather than a single dominant pathway. Within this landscape, two findings were particularly noteworthy. First, the convergence of methylation and expression evidence at the *AK4* locus suggested the presence of a potential regulatory axis linking cg06661924 methylation to *AK4* transcriptional activity. Second, several methylation signals showed subtype-specific patterns in the FinnGen dataset, particularly the clustered *VARS2* associations observed in the OTHER_SEPSIS cohort. *VARS2* encodes the mitochondrial valyl-tRNA synthetase, which participates in mitochondrial protein translation and is essential for the proper function of oxidative phosphorylation complexes (40). These observations indicate that genetic regulatory mechanisms may differ between clinically distinct sepsis subtypes. The opposite directions observed for *TOMM40L* in puerperal sepsis and neonatal bacterial sepsis further support the possibility that molecular regulatory pathways vary across sepsis phenotypes. Such heterogeneity is biologically plausible in a syndrome characterized by diverse infectious triggers and variable host responses (7, 30).

At the protein level, the evidence was comparatively limited. Only *DUT* met the predefined screening criteria in the discovery analysis, and the association remained confined to a single protein-level signal. *DUT* encodes deoxyuridine triphosphatase, an enzyme that regulates intracellular nucleotide pools and prevents uracil incorporation into DNA during replication and repair processes (41). This association showed a consistent direction of effect in both the streptococcal septicemia and pneumococcal septicemia cohorts, despite considerable uncertainty in the effect estimates, particularly for the pneumococcal septicemia cohort (95% CI 1.42-28.77). Because proteomic associations often represent downstream effects of regulatory variation, the detection of a single protein-level signal in this study may reflect the buffering effects that occur between transcriptional regulation and protein abundance.

Several aspects of the present study strengthen the interpretation of the results. By integrating multiple molecular layers, spanning an upstream epigenetic mark, its transcriptional consequence at both the bulk-tissue and immune cell-type level, and the downstream protein product, the analysis enabled the comparison of methylation, gene expression, single-cell regulatory variation, and protein abundance within a unified genetic framework (10, 11). genes supported by evidence across several of these layers were considered more likely to reflect genuine regulatory involvement in sepsis susceptibility than a signal confined to a single molecular layer. Colocalization analysis further helped distinguish loci that are more likely to share causal variants from those potentially driven by local linkage patterns (22, 25). In addition, the qPCR validation provided a clinical reference point for interpreting the genetically predicted expression effects. All twelve genes examined showed expression changes in sepsis patients that matched the direction predicted by their genetic regulation, despite the genetic and clinical datasets originating from different ancestries. As the underlying eQTL and GWAS signals were derived exclusively from European populations, this finding indicates that the predicted regulatory direction is reflected in an independent Asian clinical cohort, rather than establishing that the genetic effects themselves are of comparable magnitude between ancestries. Testing the latter will require GWAS and molecular QTL resources generated in Asian populations at comparable scale, which represents a direction for future work. The substantially smaller P values observed in the qPCR comparisons relative to the SMR estimates reflect this same distinction in what is being measured: qPCR directly compares expression levels between two clinical groups, whereas SMR estimates the effect of genetically predicted expression through an instrumental-variable framework, and the two are not statistically comparable in scale or interpretation.

Nevertheless, several limitations should be acknowledged. Most associations did not remain significant after false discovery rate correction in the discovery analysis, and therefore the reported genes should be considered prioritized candidates rather than confirmed causal factors. In addition, the validation analyses relied primarily on sepsis-related phenotypes from the FinnGen R12 release. Although the phenotype “Other septicemia” was used as the primary validation dataset, this category represents a relatively heterogeneous clinical grouping rather than a narrowly defined sepsis phenotype. It also does not capture Salmonella- or meningococcus-associated sepsis cases included in the UK Biobank discovery phenotype, for which no corresponding FinnGen endpoint was available. Together, these factors may introduce some degree of phenotypic heterogeneity and incomplete coverage into the validation analyses. While the case-ascertainment strategies of FinnGen and the UK Biobank are methodologically aligned, the Finnish population additionally reflects a distinct demographic history relative to the broader European ancestry represented in the UK Biobank, which may contribute to differences in allele frequency spectra between the two datasets. FinnGen additionally provided several pathogen- and context-specific sepsis phenotypes not available within the UK Biobank discovery dataset, offering an opportunity to examine whether the identified associations were consistent across clinically distinct presentations of this heterogeneous syndrome. Signals restricted to particular subtypes, including those observed for SLC25A35, and TOMM40L, indicate that some of the associations identified in this study may be specific to particular clinical presentations of sepsis rather than shared across all forms of the disease. As neither the UK Biobank discovery dataset nor the qPCR cohort distinguished sepsis by pathogen or clinical context, these subtype-specific signals could not be cross-checked against an equivalent stratification and warrant confirmation in future subtype-resolved studies. The QTL datasets were derived primarily from blood or circulating immune cells, which may not fully capture regulatory mechanisms occurring in organs affected during sepsis. Given that sepsis is fundamentally mediated by a systemic, immune cell-driven host response, blood-based molecular QTL data represent a biologically relevant tissue context for this syndrome; nonetheless, such data may not fully capture regulatory mechanisms specific to solid organs affected by sepsis-associated dysfunction. The eQTLGen meta-analysis in particular spans cohorts with a wide range of participant ages, which may partially dilute age-related mitochondrial gene expression effects relevant to the present biological context.

The qPCR cohort was recruited at a single center in China, reflecting the clinical setting in which the authors practice, while the genetic discovery and validation datasets were derived from European populations. The mean age of the sepsis group was approximately 30 years higher than that of the healthy control group; as mitochondrial function is known to decline with age, this age difference represents a potential confounder for the observed expression differences that was not addressed by age-matching in the present validation cohort. Disentangling age-related from sepsis-related transcriptional changes will require future validation in an age-matched clinical cohort. DNA was not collected from participants in the clinical validation cohort, precluding a direct analysis of the relationship between individual genotypes, sepsis status, disease severity, and gene expression within the same samples. Incorporating genotyping into future validation cohorts would allow this genotype-expression relationship to be examined directly at the transcriptional level, although the mQTL and pQTL layers would require distinct validation approaches, namely methylation and protein quantification assays, respectively, rather than relying on genotyping of the qPCR cohort alone. A single reference gene, ACTB, was used for RT-qPCR normalization, and no additional candidate reference genes were included in the experimental design; formal assessment of reference gene stability using multi-gene algorithms such as NormFinder or BestKeeper was therefore not performed. A previous validation study in peripheral blood mononuclear cells from septic patients identified ACTB as part of a stable reference gene combination within that population, providing indirect support for its use in the present study (42). This clinical step assessed whether the direction of genetically predicted regulation was reflected in observed gene expression during sepsis, a comparison made at the level of directional concordance rather than population-matched effect size. Extending this comparison to relative effect magnitudes across ancestries would require replicating the genetic and molecular QTL analyses in a population of Asian ancestry, for which resources of comparable statistical power are not yet available, placing such a comparison beyond the scope of the present study. Although the qPCR cohort was modest in size, the large effect sizes and adequate statistical power reported above reduce the likelihood that the observed differences reflect chance sampling variation. Correlation with SOFA score, by contrast, reached significance for only one gene, PPOX; most other genes did not show a significant relationship with disease severity in this cohort. This more limited finding may reflect the smaller effective sample size available for a continuous correlation analysis relative to a two-group comparison, and possibly population-related differences in the distribution or clinical correlates of SOFA severity between this cohort and the European populations underlying the genetic discovery analyses. Clarifying the relative contribution of these factors will require validation in larger, multi-ancestry clinical cohorts. It is also important to interpret colocalization probabilities cautiously. A low PPH4 value does not necessarily exclude the possibility of a shared signal when statistical power is limited or association signals are weak (22, 25). Finally, experimental studies will be required to determine whether the highlighted genes directly contribute to sepsis pathophysiology or instead represent markers of broader mitochondrial stress responses.

Overall, the present study suggests that genetic regulation of mitochondria-related molecular traits may contribute to sepsis susceptibility. The most consistent signals emerged when multiple molecular layers converged, particularly for *AK4* and, to a lesser extent, *SURF1*. These findings provide a prioritized set of candidates for future mechanistic studies aimed at clarifying the role of mitochondrial regulation in sepsis pathophysiology.

## Conclusions

This study identified several mitochondria-related genes and regulatory loci with genetically supported associations with sepsis susceptibility using a multi-omics SMR framework. The most consistent evidence was observed for *AK4*, which showed convergent signals across methylation and expression analyses together with supportive qPCR findings, and for *SURF1*, which displayed immune cell-specific transcriptional signals. The analysis also suggested a potential relationship between *PPOX* expression and disease severity, as indicated by its negative correlation with SOFA scores. Protein-level evidence was limited in scope, with *DUT* showing directionally consistent associations across the discovery analysis and two FinnGen subtype cohorts. Because most associations did not remain significant after multiple-testing correction in the discovery analysis, these findings should be interpreted as a prioritized set of candidate loci for future mechanistic and translational research rather than definitive causal targets.

## Data Availability

The original contributions presented in the study are included in the article/**Supplementary Material**. Further inquiries can be directed to the corresponding author.

## Supplementary material

Supplementary Figure 1 Genome-wide overview of blood eQTL-based SMR signals for prioritized mitochondria-related genes. Circos plot summarizing sepsis GWAS and blood eQTL association signals across the genomic loci of the thirteen prioritized genes. The outer track shows -log10(*P*_GWAS_) for the sepsis GWAS association at each locus; the inner track shows -log10(*P*_eQTL_) for the corresponding blood eQTL association. Purple diamonds indicate the lead cis-eQTL variant used as the instrumental variable in the SMR analysis, as reported in **Supplementary Table 6**. Gene labels are colored according to the direction of the genetically predicted association with sepsis risk (red, odds ratio > 1; blue, odds ratio < 1).

Supplementary Figure 2 Comprehensive mQTL-based SMR analysis of mitochondria-related genes. Forest plot summarizing the associations between all prioritized DNA methylation quantitative trait loci (mQTLs) and sepsis risk based on summary-data-based Mendelian randomization (SMR). Odds ratios (ORs) and 95% confidence intervals (CIs) are shown for each CpG site. Asterisks (*) indicate loci with suggestive evidence of colocalization between mQTL and sepsis GWAS signals (PPH4 > 0.5).

Supplementary Figure 3 Genetic colocalization and multi-omics convergence at the *AK4* locus. (A) Effect plot showing the SMR association between genetically predicted *AK4* expression (eQTL) and sepsis risk. (B) Effect plot showing the SMR association between genetically predicted DNA methylation at cg06661924 (mQTL) and sepsis risk. (C) Effect plot showing the SMR association between genetically predicted DNA methylation at cg10142878 (mQTL) and sepsis risk. In panels A–C, each point represents a genetic variant; the x-axis indicates the QTL effect size, and the y-axis indicates the GWAS effect size. The red triangle denotes the top cis-QTL. (D) Locus plot for the *AK4* eQTL analysis. The upper panel shows the -log_10_ (*P*_SMR-multi_) for GWAS and SMR signals across the genomic region, and the lower panel shows gene annotations. The dashed horizontal line indicates the SMR significance threshold (*P*_SMR-multi_ = 0.05). (E) Locus plot for the mQTL analyses of cg06661924 and cg10142878 at the *AK4* locus, displaying -log_10_ (*P*_SMR-multi_) for GWAS and SMR signals alongside regional gene annotations.

Supplementary Figure 4 Protein-level SMR analysis of prioritized genes. Forest plot showing the associations between genetically predicted circulating protein levels (pQTLs) and sepsis risk. A significant association was observed for deoxyuridine triphosphatase (*DUT*). Odds ratios (ORs), 95% confidence intervals (CIs), and Bayesian colocalization probabilities (PPH4) are presented.
